# Cost-Effectiveness Analysis of ^68^Ga DOTA-TATE PET/CT, ^111^In-Pentetreotide SPECT/CT and CT for Diagnostic Workup of Neuroendocrine Tumors

**DOI:** 10.3390/diagnostics11020334

**Published:** 2021-02-18

**Authors:** Matthias Frank Froelich, Moritz Ludwig Schnitzer, Adrien Holzgreve, Felix Gerhard Gassert, Eva Gresser, Daniel Overhoff, Vincent Schwarze, Matthias Philipp Fabritius, Dominik Nörenberg, Niklas von Münchhausen, Nils Große Hokamp, Christoph J. Auernhammer, Harun Ilhan, Andrei Todica, Johannes Rübenthaler

**Affiliations:** 1 Department of Radiology and Nuclear Medicine, University Medical Center Mannheim, Theodor-Kutzer-Ufer 1-3, 68167 Mannheim, Germany; Matthias.Froelich@medma.uni-heidelberg.de (M.F.F.); Daniel.Overhoff@umm.de (D.O.); Dominik.Noerenberg@medma.uni-heidelberg.de (D.N.); niklasvon.muenchhausen@umm.de (N.v.M.); 2 Department of Radiology, University Hospital, LMU Munich, Marchioninistr. 15, 81377 Munich, Germany; moritz.schnitzer14@gmail.com (M.L.S.); eva.gresser@med.uni-muenchen.de (E.G.); vincent.schwarze@med.uni-muenchen.de (V.S.); Matthias.Fabritius@med.uni-muenchen.de (M.P.F.); 3 ENETS Centre of Excellence, Interdisciplinary Center of Neuroendocrine Tumors of the GastroEnteroPancreatic System (GEPNET-KUM), LMU Munich, 81377 Munich, Germany; Adrien.Holzgreve@med.uni-muenchen.de (A.H.); christoph.auernhammer@med.uni-muenchen.de (C.J.A.); harun.ilhan@med.uni-muenchen.de (H.I.); Andrei.Todica@med.uni-muenchen.de (A.T.); 4 Department of Nuclear Medicine, University Hospital, LMU Munich, Marchioninistr. 15, 81377 Munich, Germany; 5 Department of Diagnostic and Interventional Radiology, Klinikum rechts der Isar, Technical University of Munich, Ismaninger Str. 22, 81675 Munich, Germany; felix.gassert@tum.de; 6 Institute for Diagnostic and Interventional Radiology, University Hospital Cologne, 50937 Cologne, Germany; nils.grosse-hokamp@uk-koeln.de; 7 Department of Internal Medicine 4, University Hospital, LMU Munich, 81377 Munich, Germany

**Keywords:** neuroendocrine tumors, nuclear imaging, ^68^Ga-DOTA-TATE PET/CT, cost-effectiveness

## Abstract

Neuroendocrine tumors (NETs) are relatively rare neoplasms arising from the hormone-producing neuroendocrine system that can occur in various organs such as pancreas, small bowel, stomach and lung. As the majority of these tumors express somatostatin receptors (SSR) on their cell membrane, utilization of SSR analogs in nuclear medicine is a promising, but relatively costly approach for detection and localization. The aim of this study was to analyze the cost-effectiveness of ^68^Ga-DOTA-TATE PET/CT (Gallium-68 DOTA-TATE Positron emission tomography/computed tomography) compared to ^111^In-pentetreotide SPECT/CT (Indium-111 pentetreotide Single Photon emission computed tomography/computed tomography) and to CT (computed tomography) alone in detection of NETs. A decision model on the basis of Markov simulations evaluated lifetime costs and quality-adjusted life years (QALYs) related to either a CT, SPECT/CT or PET/CT. Model input parameters were obtained from publicized research projects. The analysis is grounded on the US healthcare system. Deterministic sensitivity analysis of diagnostic parameters and probabilistic sensitivity analysis predicated on a Monte Carlo simulation with 30,000 reiterations was executed. The willingness-to-pay (WTP) was determined to be $ 100,000/QALY. In the base-case investigation, PET/CT ended up with total costs of $88,003.07 with an efficacy of 4.179, whereas CT ended up with total costs of $88,894.71 with an efficacy of 4.165. SPECT/CT ended up with total costs of $89,973.34 with an efficacy of 4.158. Therefore, the strategies CT and SPECT/CT were dominated by PET/CT in the base-case scenario. In the sensitivity analyses, PET/CT remained a cost-effective strategy. This result was due to reduced therapy costs of timely detection. The additional costs of ^68^Ga-DOTA-TATE PET/CT when compared to CT alone are justified in the light of potential savings in therapy costs and better outcomes.

## 1. Introduction

Neuroendocrine tumors (NETs) are rare malignancies arising from the hormone-producing neuroendocrine system [1]. Determined by the distribution of neuroendocrine cells in the body, the common sites of NETs are lung with 30.6%, small intestine with 22.2%, rectum with 16.2%, colon with 13.4% and pancreas with 10.8%. It also can rarely occur in stomach, ovaries, thyroid and the adrenal glands [2]. Even though most of NETs present as well-differentiated and slow-growing tumors, metastatic spreading in liver, lymph nodes and in bones is common [3]. The most common site of metastasis is the liver as 60% to 80% of patients present with or develop liver metastases during their illness [4]. This is due to the often-unspecific initial presentation [2]. However, over recent decades the incidence of NETs has been increasing and currently is reported to be between 3.6 and 5.9 per 100,000 inhabitants [2,4,5]. According to data from the US Surveillance Epidemiology and End Results database, neuroendocrine neoplasms may be even more prevalent than hepatobiliary, esophageal and pancreatic adenocarcinomas all together. The increase in annual incidence of neuroendocrine neoplasms is not only noted in the USA but also in Australia, in European countries, e.g., in Norway, as well as in Asian countries, e.g., in Taiwan [5,6]. This rise is related both to an increasing awareness regarding the disease and to improving diagnostic procedures, including imaging techniques [7]. However, currently neuroendocrine neoplasms are still too rare overall to be accessible for screening methods or preventative measures [5]. Overall median survival of NET patients varies between 41 months and 75 months, depending on prognostic factors such as tumor stage, tumor grade and tumor site. During their illness, patients often struggle with symptoms caused by the surplus production of peptides and hormones such as serotonin [1]. These metabolically active substances can be reason for patients to present themselves to a physician, but the majority of neuroendocrine tumors are non-functioning [8], although the resulting “carcinoid syndrome” of metabolically functional neuroendocrine tumors with an incidence of 19% is a common sign of the disease and is associated with flushing and diarrhea [1,9]. Other potential diseases associated with NETs are Zollinger–Ellison syndrome, hypoglycemia and bronchospasm [10]. The term of neuroendocrine carcinoma (NEC) has also been proposed for the poorly differentiated neuroendocrine neoplasms (NEN), as opposed to the well-differentiated neuroendocrine tumors (NET), and efforts are being made to uniformize the nomenclature, e.g., by the International Agency for Research on Cancer—World Health Organization (IARC-WHO) [11]. Yet the term “NET” is still widely used as a blanket term for all entities of neuroendocrine neoplasms. In the currently largely incurable metastatic stage, improvement of symptoms is the focus of care in patients with metastatic NET [12]. NET patients without metastatic disease are potentially curable through surgical intervention, but at a considerable risk of relapse [1,4].

For planning and managing further interventions and treatment procedures, precise and comprehensive imaging is crucial [3]. In clinical routine, computed tomography (CT), ultrasound (US), magnetic resonance imaging (MRI), single-photon emission computed tomography (SPECT/CT) and positron emission tomography (PET/CT) are the standard of diagnostic procedures [3]. For many years, spiral CT was considered the gold-standard imaging technique for detecting NETs, as it offers many advantages in diagnostic workup. Wide availability, low costs and short examination duration are advantages of CT. Additionally, due to technical progress the radiation dose of an examination could be reduced significantly [8]. SPECT/CT is also a considerable diagnostic modality for detecting NETs and for following diagnostic workup. SPECT/CT is an increasingly used nuclear medical imaging method as a hybrid modality between CT and SPECT in a single examination [13]. Since 1999, SPECT/CT has been used in clinical routine starting with a dual-headed sodium iodide crystal gamma camera combined with a low-dose CT and has developed since then [14]. This imaging method offers many advantages in diagnostics as the radioactive tracers that are used to detect malignancies in the body have a relatively long half-life compared to tracers used in PET/CT [15]. For instance, SPECT/CT imaging using tracers labeled with the radioligand Indium-111 has traditionally and successfully been used in patients with neuroendocrine neoplasms. The major dissimilarity between PET/CT and SPECT/CT relies on the emission of the different radiotracers. Whereas PET/CT scanners measure emitted positrons, SPECT/CT images are created by measuring emitted gamma rays [16]. This is a main distinction criterion compared to CT as they do not only offer morphological information about the tissue, but additionally functional information that may simplify the characterization of tumor lesions as the highlighted regions show an enlarged uptake of the used tracer f.e. somatostatin analogs. Further, the availability of SPECT/CT imaging devices is better than with PET/CT. Over recent years, somatostatin analogs are gaining more importance in the diagnostic workup of NET patients as 70% to 90% of neuroendocrine tumor cells express somatostatin receptors on their cell membrane [3]. There are five different types of somatostatin receptors on human cell membranes detected so far with the appellations SSTR1-5/SST_1–5_ [17]. For NET imaging, the most prevalent and thus most interesting receptor subtype is the SSTR2 with an estimated prevalence of 70% [18,19]. PET/CT imaging using SSR targeted tracers such as ^68^Ga-DOTA-TATE (Gallium-68-DOTA-TATE), ^68^Ga-DOTA-TOC and ^68^Ga-DOTA-NOC has been increasingly implemented in disease management of patients with neuroendocrine neoplasms owing to its superior performance compared to conventional imaging in initial detection, staging, detection of recurrence and unknown primary tumor as well as for its intriguing capability to evaluate patients for radioligand therapy in a theragnostic setting [20,21]. Hence, several studies made the diagnostic sensitivities and specificities of somatostatin analogs in PET/CT and in SPECT/CT the subject of discussion and compared the advantages of both modalities comprehensively [22,23,24,25,26]. As a result of an extensive investigation of this field, clinical guidelines mainly recommend using ^68^Ga-DOTA-TATE PET/CT over somatostatin receptor scintigraphy methods for tumor staging, preoperative imaging and restaging [8]. The increasing role of PET/CT imaging for neuroendocrine neoplasms, especially using SSR targeted tracers, is generally reflected by an increasing implementation and appreciation of PET/CT imaging in national and international guidelines on NETs [9,27,28]. Yet high diagnostic costs and the resulting financial burden in the context of the increasing incidence of NETs have increased the need for an additional evaluation of the diagnostic modalities from a cost-effectiveness perspective [10]. In our analysis we focus on two of the most common and widely available tracers for each modality: ^68^Ga-DOTA-TATE for PET/CT imaging and ^111^In-pentetreotide for SPECT/CT imaging. Although MRI is regarded as an integral part of clinical imaging in NET, it was excluded from the analysis as published literature often focuses on comparison of CT and functional imaging such as SPECT/CT and PET/CT. 

Therefore, in view of the growing clinical importance of neuroendocrine neoplasms with an increasing incidence on the one hand and increasingly sophisticated but expensive imaging procedures on the other, the aim of this study was to analyze the cost-effectiveness of CT, ^111^In-pentetreotide SPECT/CT and ^68^Ga-DOTA-TATE PET/CT for detecting neuroendocrine tumors based on the US healthcare system.

## 2. Materials and Methods

### 2.1. Model Structure

**Figure 1:**
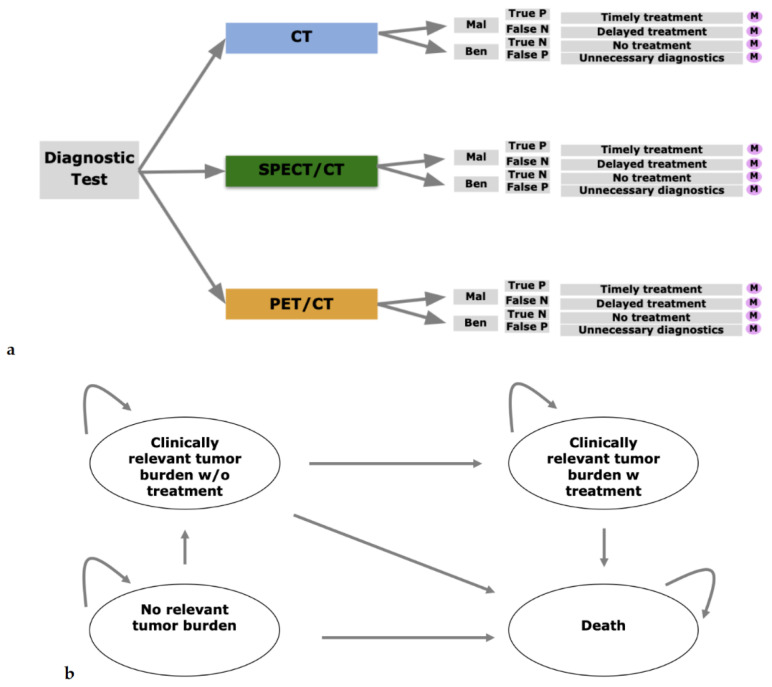
(**a**) Model overview: a decision model for strategies CT, SPECT/CT and PET/CT. For every outcome, a Markov model analysis was carried out. (**b**) Markov model with potential states “no relevant tumor burden”, “clinically relevant tumor burden w/o treatment”, “clinically relevant tumor burden with treatment” and “death”. The first state was determined depending on the outcomes in the decision model.

To evaluate the cost-effectiveness of 68Ga-DOTA-TATE PET/CT compared to CT and 111In-pentetreotide SPECT/CT, a decision model on the basis of a decision-analytic software (TreeAge Pro Version 19.1.1, Williamstown, MA, USA) was designed. For evaluation of patients’ long-term outcomes, a Markov model was applied. A Markov model is defined by being a stochastic model for estimation of complex changing systems used to model the probabilities of different health-related states and the transition rates among these. In general, future health-related states are assumed to be independent from previous states. In our model, we chose the starting point depending on the results of diagnostic examination. For instance, TP equals the state of a relevant tumor burden with treatment, whereas a FN equals the state of a clinically relevant tumor burden without treatment. Patients with a true negative (TN) or a false positive (FP) state are ranked as no relevant tumor burden. Of course, these were adjusted for diagnostic costs dependent on the diagnostic strategy. The model and its different stages are summarized in Figure 1a,b. The Markov model developed for this analysis consists of the states “no relevant tumor burden”, “clinically relevant tumor burden w/o treatment”, “clinically relevant tumor burden with treatment” and “death”. The starting state for the model was defined in the following fashion. In case of a true positive, “clinically relevant tumor burden with treatment” was defined as the starting state. In case of a false negative, “clinically relevant tumor burden without treatment” was defined as starting state. In both true negative and false positive patients, “no relevant tumor burden was defined as starting state. Corresponding input parameters in terms of costs, quality of life and transition probabilities are defined below. To further enhance the understanding of the behavior of the Markov model, we added a Supplementary Figure S1 depicting the development dependent on the initial diagnostic outcome. All Markov model input parameters are summarized in Table 1.

### 2.2. Input Parameters

An average patient age of 30 years was assumed according to published research [29]. The discount rate was determined to be 3.00%. Further, willingness-to-pay (WTP) was determined as $100,000 per quality-adjusted life year (QALY) according to recommendations of published research specializing in performing cost-effectiveness analyses [27,36,37,38]. Additionally, pre-test probability of NET was determined to be 5% according to recent literature and refers to a setting where NET lesions are suspected and therefore imaging with either CT, SPECT/CT or PET/CT is indicated [29]. Our analysis is based on the US healthcare system in year 2020. Age-specific risk of death was derived from the US Life Tables as the largest available data set. A summarized overview is shown on Table 1.

### 2.3. Efficacy of Treatment Modalities

CT sensitivity and specificity were set to 77% and 86%, sensitivity and specificity of 68Ga-DOTA-TATE PET/CT and 111In-pentetreotide SPECT/CT were set to 91% and 92%, and 70% and 96%, respectively [8]. 

### 2.4. Costs and Utilities

Treatment costs of CT, PET/CT, SPECT/CT and biopsy costs were collected from Medicare in 2020. In addition to that, the costs for treatment, surgery and long-term healthcare costs were included in the analysis [4]. 

Utilities were raised as quality-adjusted life years (QALYs) as the value of the patient’s health state [28,30,31]. 

### 2.5. Transition Probabilities

In accordance with the previously described Markov model, probabilities of death with timely and delayed treatment were considered in the analysis [32,33]. Further, probabilities of disease progression or recurrence, death for a competitive cause and death without treatment were added to the analysis [2,37,38]. Additionally, for the estimation of probability of death for other reasons, US Life Tables were utilized.

### 2.6. Cost-Effectiveness Analysis

According to the described WTP and discount rate, the expected QALYs and costs were calculated for a baseline scenario. Furthermore, the ICER as a tool to prove cost-effectiveness was assessed. 

Definitions:

Willingness to pay (WTP): In economic analyses related to healthcare systems, a WTP limit stands for an estimated limitation of expense for a certain health benefit a healthcare system is willing to pay.

Incremental cost effectiveness ratio (ICER): The ICER is a calculated parameter that indicates the economic value of a number of different comparable strategies. The ICER is calculated by the following formula:

(1) $$\mathrm{ICER}=\frac{\left(C_{1}-C_{0}\right)}{\left(E_{1}-E_{0}\right)}$$

with C_1_ and E_1_ and C_0_ and E_0_ indicating cost and effect of the one strategy and effect of the compared strategy, respectively. The result constitutes the additional expense one approach has compared to another approach per QALY. 

Sensitivity analysis: A sensitivity analysis is performed to determine how the variation of an input parameters’ value has an impact on a dependent variable. Thus, a sensitivity analysis should assess the uncertainty of a model. If the result of the ICER calculation shows only minimal variations by these manipulations, the ICER can be regarded as robust whereas big variations indicate a higher grade of uncertainty of the analysis. 

Deterministic sensitivity analysis: In deterministic sensitivity analysis, certain (one-way sensitivity analysis) or multiple (multivariate sensitivity analysis) input parameters are modified within a range. 

Probabilistic sensitivity analysis: In a probabilistic sensitivity analysis, variable parameters are sampled from their respective distributions rather than assigning a point estimate value. Thus, the analysis of a model is performed with a larger number of iterations to figure out potential variations of results.

Cost-effectiveness acceptability curve: A cost-effectiveness acceptability curve is a graphical method to depict the relationship between ICER and a cost-effectiveness threshold within a certain range showing the impact of uncertainty on the result of an economical evaluation.

**Figure 2:**
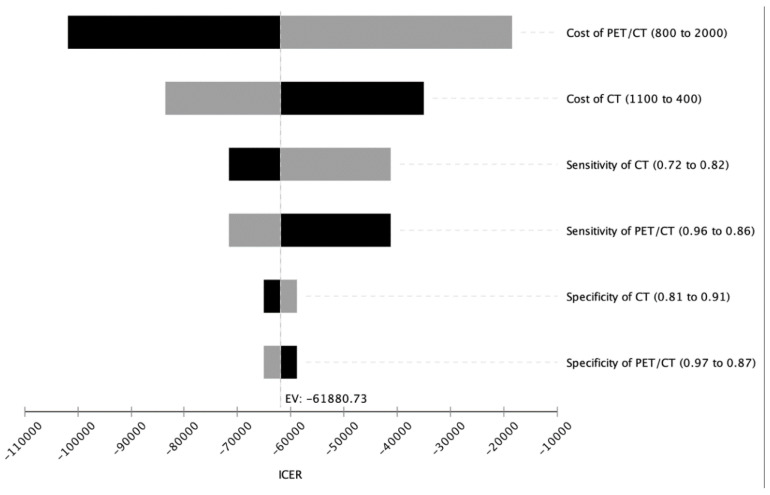
Deterministic sensitivity analysis tornado diagram.

In our analysis investigating the cost-effectiveness of NET imaging methods, the discount rate was set to 3.00% and the WTP to $100,000 per QALY. For the cost-effectiveness account, the expected costs were calculated in United States dollars (USD) and according QALYs were calculated. ICERs were calculated and analyzed to compare the different imaging strategies. A deterministic sensitivity analysis was carried out to investigate the influence of various input parameters on the model. To visualize our results, all outcomes are displayed in a tornado diagram in Figure 2. 

### 2.7. Sensitivity Analysis

To analyze the robustness of the model, deterministic and probabilistic sensitivity analyses were performed. For the latter, a number of 30,000 Monte Carlo iterations were applied. Based on the probabilistic analysis, acceptability curves were estimated. To allow for comprehensive analysis of the topic, we performed additional sensitivity analyses comparing 68Ga-DOTA-TATE PET/CT and CT (Figure S2). Furthermore, the willingness to pay per QALY may vary significantly between several European countries. To analyze the impact of WTP, we added an additional sub-analysis of our probabilistic sensitivity analysis which is visualized as an acceptability curve (Figure S3).

## 3. Results

### 3.1. Estimated Outcomes and Corresponding Costs

Results were measured in a Markov model. Therefore, patients without required treatment (true negative and false positive), patients with a timely surgery and treatment (true positive) and patients with a delayed surgery and treatment (false negative) were modeled comparably. After a year, true positive patients had expected cumulative costs of $146,443.00 and a cumulative quality of life of 0.768 QALYs. In comparison, the group with false negative finding had expected cumulative costs of $188,977.00 and a cumulative quality of life of 0.612 QALYs. Furthermore, patients without initially indicated treatment showed a cumulative quality of life of 1 and 0.779 and cumulative costs of $23,979.07 and $34,208.07 for true negative (TN) and false positive (FP) cases, respectively. 

### 3.2. Cost-Effectiveness Analysis

**Figure 3:**
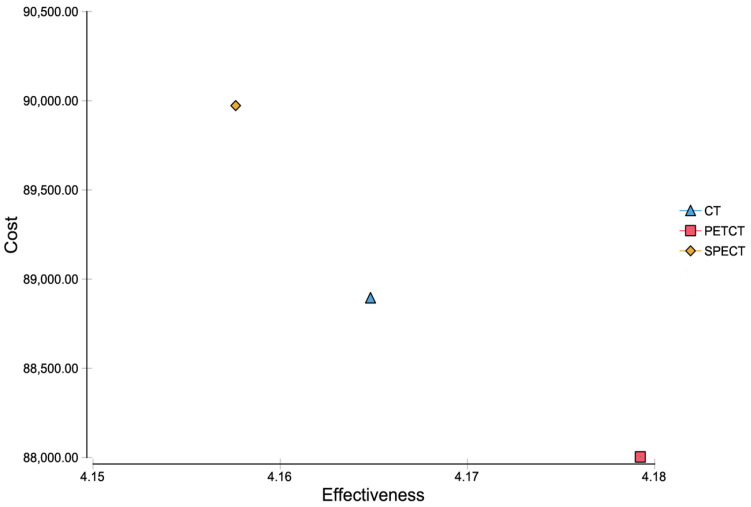
Cost-effectiveness analysis.

Taking into account the results from the Markov model, a baseline cost-effectiveness analysis for CT led to summed up costs of $88,894.71 and an efficacy of 4.165 QALYs. SPECT/CT led to summed up costs of $89,973.34 and an efficacy of 4.158, whereas PET/CT let to summed up costs of $88,003.07 with an efficacy of 4.179 (Figure 3). Consequently, the corresponding incremental cost-effectiveness ratio (ICER) of PET/CT was negative.

### 3.3. Deterministic Sensitivity Analysis

A deterministic sensitivity analysis including costs, sensitivities and specificities of all diagnostic modalities was carried out to inspect the validity of our analyzed model. For sensitivity, specificity and the three imaging modalities in the range of the assumed baseline values were examined. The ICER for PET/CT remained below the willingness-to-pay (WTP) limit of $100,000 per QALY in the predicted ranges indicating cost-effectiveness of PET/CT (Figure 2).

### 3.4. Probabilistic Sensitivity Analysis

**Figure 4:**
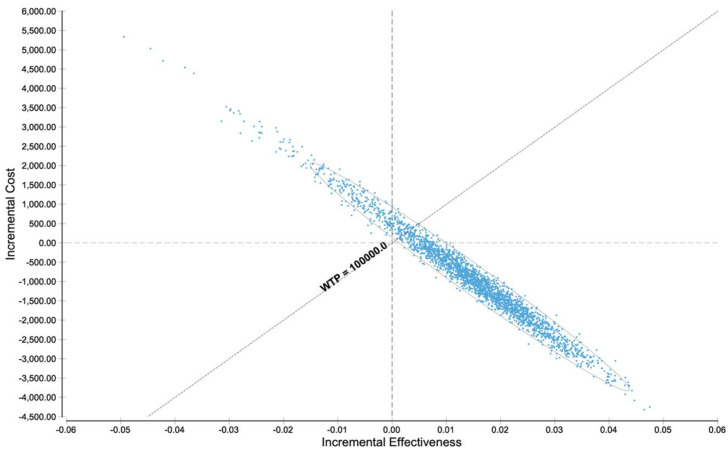
Probabilistic sensitivity analysis utilizing Monte Carlo simulations with 30,000 reiterations: incremental cost-effectiveness scatterplot of ^68^GaDOTA-TATE PET/CT compared to CT.

To further inspect the validity of the analyzed model, a probabilistic sensitivity analysis of the basis of the distributions described in Table 1 was carried out. At the WTP of $100,000 per QALY, PET/CT was cost-effective in a major part of reiterations (Figure 4). Additional sensitivity analyses proved that PET/CT is the cost-effective strategy in a major part of reiterations among a wide range of PET/CT and CT costs (Figure S2) and in a range from 0 to $200,000 WTP per QALY (Figure S3).

## 4. Discussion

This study demonstrates that ^68^Ga-DOTA-TATE PET/CT can be a cost-effective imaging strategy for detecting neuroendocrine tumors in comparison to ^111^In-pentetreotide SPECT/CT and CT alone. These results are consistent with the current guidelines for the standard of care in neuroendocrine tumors, recommending ^68^Ga-DOTA-TATE PET/CT to be used for tumor staging, restaging and preoperative imaging planning as it is best suited for the majority of NETs and their metastases in liver, bone and lymph nodes due to its higher sensitivity [8].

**Figure 5:**
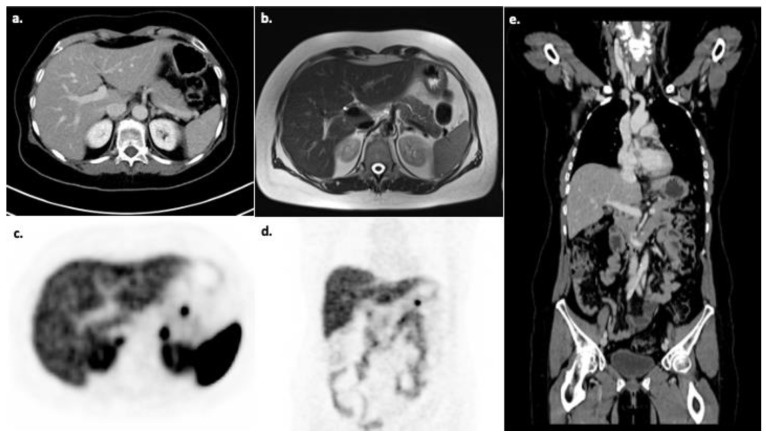
Patient from our institution with clinically suspected neuroendocrine tumor (NET). ^68^Ga-DOTA-TATE PET/CT imaging and MRI were performed for diagnostic work-up. (**a**) CT in venous phase is unremarkable and shows homogeneous liver parenchyma as well as normal pancreatic parenchyma. (**b**) T2-weighted MRI of the same patient also shows homogeneous liver parenchyma and normal pancreatic parenchyma with no clear evidence of malignancy. (**c**) ^68^Ga-DOTA-TATE PET in the axial plane reveals a markedly elevated somatostatin receptors (SSR) expression in line with a small tumor in the pancreatic tail in line with diagnostic PET features of a NET. Physiological SSR expression of the adrenal glands can also be noted. (**d**) ^68^Ga-DOTA-TATE PET in the coronal plane visualizes the NET tumor clearly and the anatomical position of the tumor in the pancreatic tail. (**e**) Coronal CT imaging can be used for further planning of potential surgical treatment, although the tumor cannot be visualized solely relying on CT imaging.

In Figure 5, the diagnostic workup with ^68^Ga-DOTA-TATE PET/CT, CT and MRI of a patient from our institution with clinically suspected NET is shown. In this case, a small neuroendocrine tumor in the pancreatic tail with markedly increased SSR expression is shown which can only be seen in ^68^Ga-DOTA-TATE PET/CT, whereas the CT and MRI alone did not show any evidence of a tumor. This case exemplifies the diagnostic power and the high sensitivity of ^68^Ga-DOTA-TATE PET/CT for the detection of neuroendocrine neoplasms compared to CT and MRI alone. Further, lesions that were able to be detected in common CT and MRI examinations cannot always be characterized clearly in tumor entity and therefore are at the beginning of the diagnostic workup classified as an incidentaloma. As the functional imaging offered by radioligand tracers may allow a more specific characterization of the lesion, an additional PET/CT or SPECT/CT is a useful strategy for further diagnostic workup. An overview of the Markov model analysis performed in the current study is given in Figure 1. The results of the cost-effectiveness analysis are shown in Figure 3 and clearly demonstrate, in a nutshell, that ^68^Ga-DOTA-TATE PET/CT exhibits the most profitable combination of low costs and high effectiveness. Figure 2 and Figure 4 in detail illustrate the superiority of ^68^Ga-DOTA-TATE PET/CT when compared to CT in terms of sensitivity and incremental cost-effectiveness.

In a broader sense, the distribution of resources in a healthcare system cannot entirely be founded on terms of medical effectiveness. It is also essential for an economically working healthcare system to include a judgement grounded on the cost-effectiveness of a potential treatment strategy. Relating to this analysis, the justification of an effective but costly PET/CT examination is questioned in order to provide a resource-saving policy in the long term. Therefore, cost-effectiveness analyses are necessary to offer a tool for decision makers in healthcare policy to decide which treatment to use or to avoid. The results of cost-effectiveness analyses as in this analysis deliver far more comprehensive data than clinical trials alone, simplifying the decisions of a healthcare provider [39]. Nonetheless, there are still concerns about the capability of cost-effectiveness analyses to address this matter [40]. In particular, the ICER as a useful indicator for cost-effectiveness is doubted to be useful for reducing ineffective treatment types. It must be underlined that cost-effectiveness analyses are a tool to inform and to measure the effectiveness as a whole, but there is no guarantee that the results entail a superior decision in every singular individual case. Nevertheless, we highly trust in the effectiveness of such analyses as a way to improve diagnostics, as only the best suited diagnostic device is used for the patient not only regarding its initial cost, but the long-term effectiveness of the outcome. This shift in healthcare policy may have a substantial influence on the patients’ outcomes and quality of life in the long term.

PET/CT has been shown to be a powerful and cost-effective imaging modality, and not only for neuroendocrine tumors. For instance, from a cost perspective view ^68^Ga PSMA PET/CT (^68^Ga Prostate-Specific Membrane Antigen PET/CT) seems to be superior to extended pelvic lymph node dissection in patients with prostate cancer [41], while ^18^F-FDG PET-CT seems to be superior to CT alone for preoperative evaluation of patients with monometastatic non-small-cell lung cancer (NSCLC) [42], as well as seeming to be cost-effective in the long term for the management of patients with locally advanced head and neck cancer.

The results of our current investigation regarding the cost effectiveness of ^68^Ga-DOTA-TATE PET/CT, ^111^In-pentetreotide SPECT/CT and CT for the detection of neuroendocrine tumors are in line with previously published data. In 2011, an analysis compared costs of ^111^In-DTPA-octreotide SPECT/CT and ^68^Ga-DOTATOC PET/CT for staging of entero-pancreatic NETs. In summary, the authors concluded that SSR targeted PET/CT was significantly more cost-effective due to lower costs and fewer additional required examinations compared to ^111^In-DTPA-octreotide SPECT/CT [43]. As other studies show as well [44], these data further indicate that the higher cost-effectiveness of SSR targeted PET/CT does not seem to depend on the tracer chosen, as both ^68^Ga-DOTA-TATE and ^68^Ga-DOTA-TOC have been analyzed so far and both have shown to be superior to SPECT/CT imaging and/or CT imaging alone.

In sum, from an economical point of view, SSR targeted PET/CT imaging such as ^68^Ga-DOTA-TATE PET/CT can be considered as a cost-effective strategy for the imaging of neuroendocrine tumors for G1 and G2 NETs.

### Limitations

Although our model proves that ^68^Ga-DOTA-TATE PET/CT appears to be the cost-effective modality for NET diagnosis, some limitations need to be acknowledged and taken into consideration. Basically, our study was designed for evaluating the cost-effectiveness of ^68^Ga-DOTA-TATE PET/CT, ^111^In-pentetreotide SPECT/CT and CT based on the US healthcare system. Consequently, we need to acknowledge that our results do not provide sufficient informative value to assess the cost-effectiveness of these modalities in other healthcare systems. Even though the majority of NET cells have a utilizable expression of somatostatin receptors, some tumor cells apparently express a smaller amount of SSRs. Additionally, some small and undifferentiated tumors are hardly visible in this imaging method. Both may lead to false negative results [3]. In addition to that, the pancreas is known to harbor sites of physiological SSR expression, especially in the uncinate process, which complicates an accurate differentiation between tumor lesions and healthy tissue in NETs with a primary pancreatic site [3]. As described above, MRI was consciously excluded from this analysis, nonetheless, NETs are very variable in their tumor sites and entities and therefore there is no one-size-fits-all solution. Still, there may be some tumor subgroups where other diagnostic modalities are superior to the standard protocol. For instance, patients with multiple endocrine neoplasia type 1 (MEN1) do not require such extensive nuclear medical imaging and would benefit from an abbreviated examination protocol. Verde et al. 2020 proved that MEN1 patients may profit from avoiding contrast medium administration as a shortened MRI examination limited to diffusion weighted imaging (DWI) and T2-w fat suppressed sequences offer a more precise detection of pancreatic NET lesions [45]. In the end—besides any cost-effectiveness—it is a case-by-case decision which modality fits best for the present case. Besides that, even though ^68^Ga-DOTA-TATE PET/CT is recommended for most types of NET lesions in the ENETS Guidelines, ^18^F-FDG PET/CT is already the standard of care for G3 and high G2 NETs due to their higher glucose metabolism and lack of SSR expression [8], especially since G3 NEC usually do not express SSR on their cell membrane and ^68^Ga-DOTA-TATE PET/CT might not be the suitable imaging modality of choice for diagnostic work-up. Therefore, for G3 and high G2 NETs ^18^F-FDG PET/CT might be the preferable diagnostic modality of choice due to a potentially higher value of prognostic information [8]. It must be emphasized that our study does not distinguish between different tumor stages or gradings, and consequently the results of the cost-effectiveness calculation for each grade may differ due to a divergence of quantity of SSR expression. Thus, our analysis is limited to tumor grades G1 and G2 with a sufficient amount of SSR expression on their cell membrane, as those are the targets for somatostatin analog imaging and also potential therapy.

## 5. Conclusions

In conclusion, our study demonstrated the cost-effectiveness of ^68^Ga-DOTA-TATE PET/CT for detecting neuroendocrine tumors (NETs) compared to ^111^In-pentetreotide SPECT/CT and to CT alone. In daily clinical practice, ^68^Ga-DOTA-TATE PET/CT can be considered the most economical approach for diagnostic workup of neuroendocrine tumors. 

## Figures and Tables

**Table 1:** Input parameters.

Name	Estimate	Distribution	Source
Pre-test probability of NET	5%	β	Dasari et al. 2017 [29]
Expected age at diagnosis	30		Dasari et al. 2017 [29]
Assumed WTP/ QALY	$100,000.00		Sanders et al. 2016 [27]
Discount rate	3.00%		Sanders et al. 2016 [27]
**Diagnostic test performances**			
CT sensitivity	77%	β	Sundin et al. 2017 [8]
CT specificity	86%	β	Sundin et al. 2017 [8]
SPECT/CT sensitivity	70%	β	Sundin et al. 2017 [8]
SPECT/CT specificity	96%	β	Sundin et al. 2017 [8]
PET-CT sensitivity	91%	β	Sundin et al. 2017 [8]
PET-CT specificity	92%	β	Sundin et al. 2017 [8]
**Costs (Acute)**			
CT	$787	γ	Medicare (74,177 + 71,260 + 70,491)
PET/CT	$1375.00	γ	Medicare (78814)
SPECT/CT	$1242.00	γ	Medicare (78,803 + 74,177 + 71,260 + 70,491)
Biopsy	$1375.00	γ	Medicare (48,102)
Timely surgery + treatment (true positive)	$85,068.00	γ	Medicare/Expert opinion
Delayed surgery + treatment (false negative)	$127,602.00	γ	Medicare/Expert opinion
Unnecessary biopsy (false positive)	$1375.00	γ	Medicare (36,246)
No further action required (true negative)	$0.00	γ	Assumption
**Costs (long term)**			
Yearly costs without relevant tumor burden	$0.00	γ	Assumption
Yearly costs with/after treated NET	$2107.00	γ	Spolverato et al. 2015 [4]
Yearly costs with/after treated NET (clinically relevant)	$61,375.00	γ	Spolverato et al. 2015 [4]
**Utilities**			
Health state utility values of NET patients without relevant tumor burden	1	β	Assumption
Health state utility values of NET patients (no clinically relevant tumor burden)	0.779	β	Chua et al. 2018 [28]
Health state utility values of NET patients (clinically relevant tumor burden)	0.768	β	Casciano et al. 2012 [30]
Health state utility values of NET patients with disease progression	0.612	β	Casciano et al. 2012 [30]
Health state utility values of NET patients without treatment	0.690	β	Teunissen et al. 2004 [31]
Death	0		Assumption
**Transition probabilities**			
Risk of death with timely treatment	4.00%	β	Korse et al. 2013 [32]
Risk of death with delayed treatment	4.50%	β	Keizer et al. 2016 [33]
Risk of death with no treatment	12.12%	β	Man et al. 2018 [2]
Risk of death for a competitive cause	2.50%	β	Low et al. 2019 [34]
Risk of disease progression/recurrence	8.80%	β	Ter-Minassian et al.2013 [35]
Risk of death for other reason	Life Table x relative Risk	β	US Life Table
Clinical rel w/o treatment to clin rel with treatment	1	β	Assumption

## Data Availability

No new data were created or analyzed in this study. Data sharing is not applicable to this article.

## Supplementary Materials

The following are available online at https://www.mdpi.com/2075-4418/11/2/334/s1, Figure S1: Modeling results of Markov model based on diagnostic outcomes. Note that the distribution between the starting situations (TP, FN, TN, FP) depends on the sensitivity and specificity of each diagnostic modality. Figure S2: Sensitivity analysis for multiple cost thresholds of CT and PET-CT based on a WTP threshold of $100,000 per QALY. All numbers in US$, Figure S3: Proportion of iterations cost-effective for different WTP thresholds stratified for all three diagnostic strategies.
