# Supplementary figures and images for: Cost-Effectiveness Analysis of 68Ga DOTA-TATE PET/CT, 111In-Pentetreotide SPECT/CT and CT for Diagnostic Workup of Neuroendocrine Tumors
Source: Diagnostics (Basel). 2021 Feb 18;11(2):334. doi: 10.3390/diagnostics11020334 (PMC7922846; doi:10.3390/diagnostics11020334)

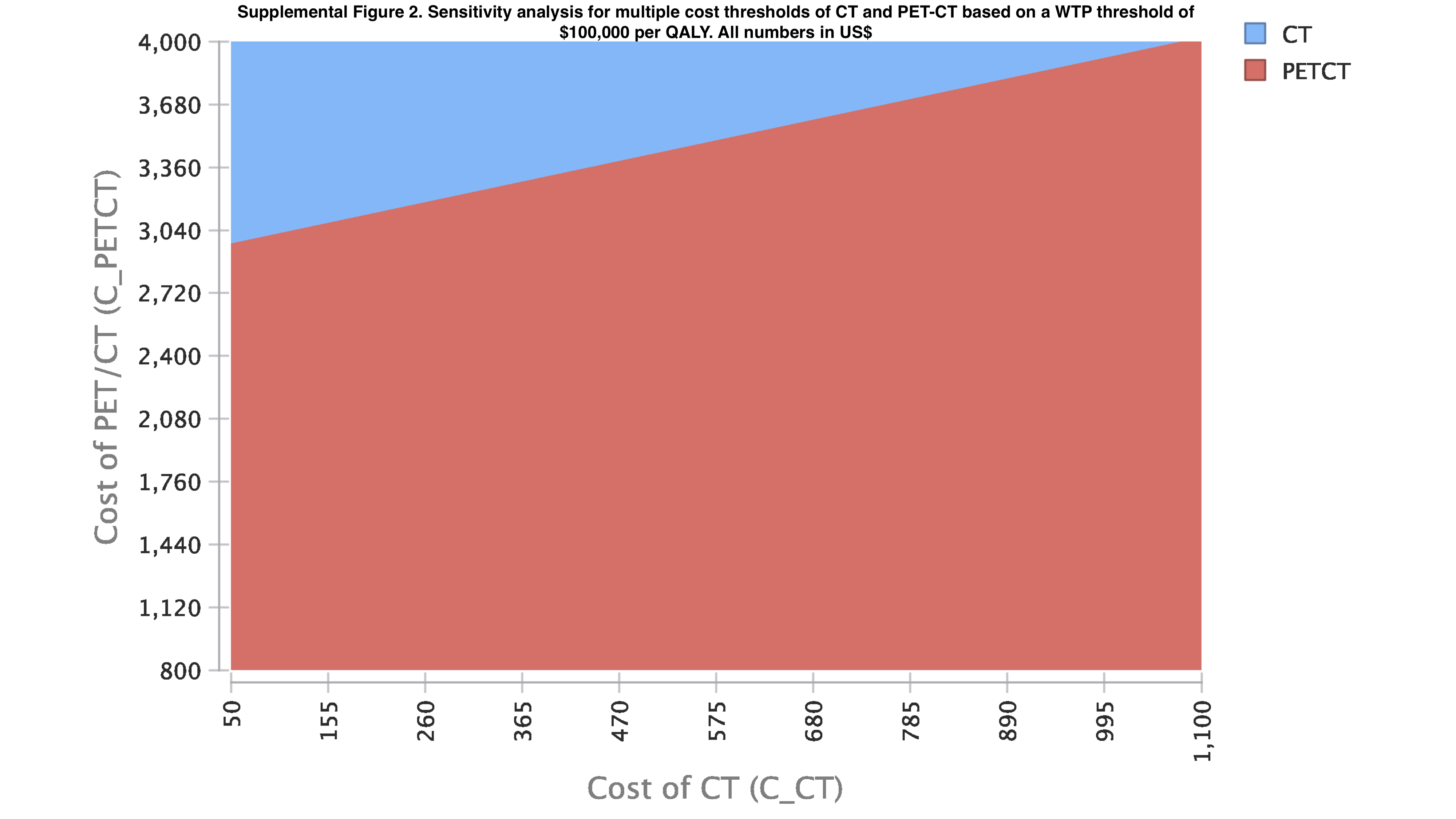

Supplement: Supplementary file 1 [file diagnostics-11-00334-s001.zip › Figure 2 Supplemental NET.tiff]

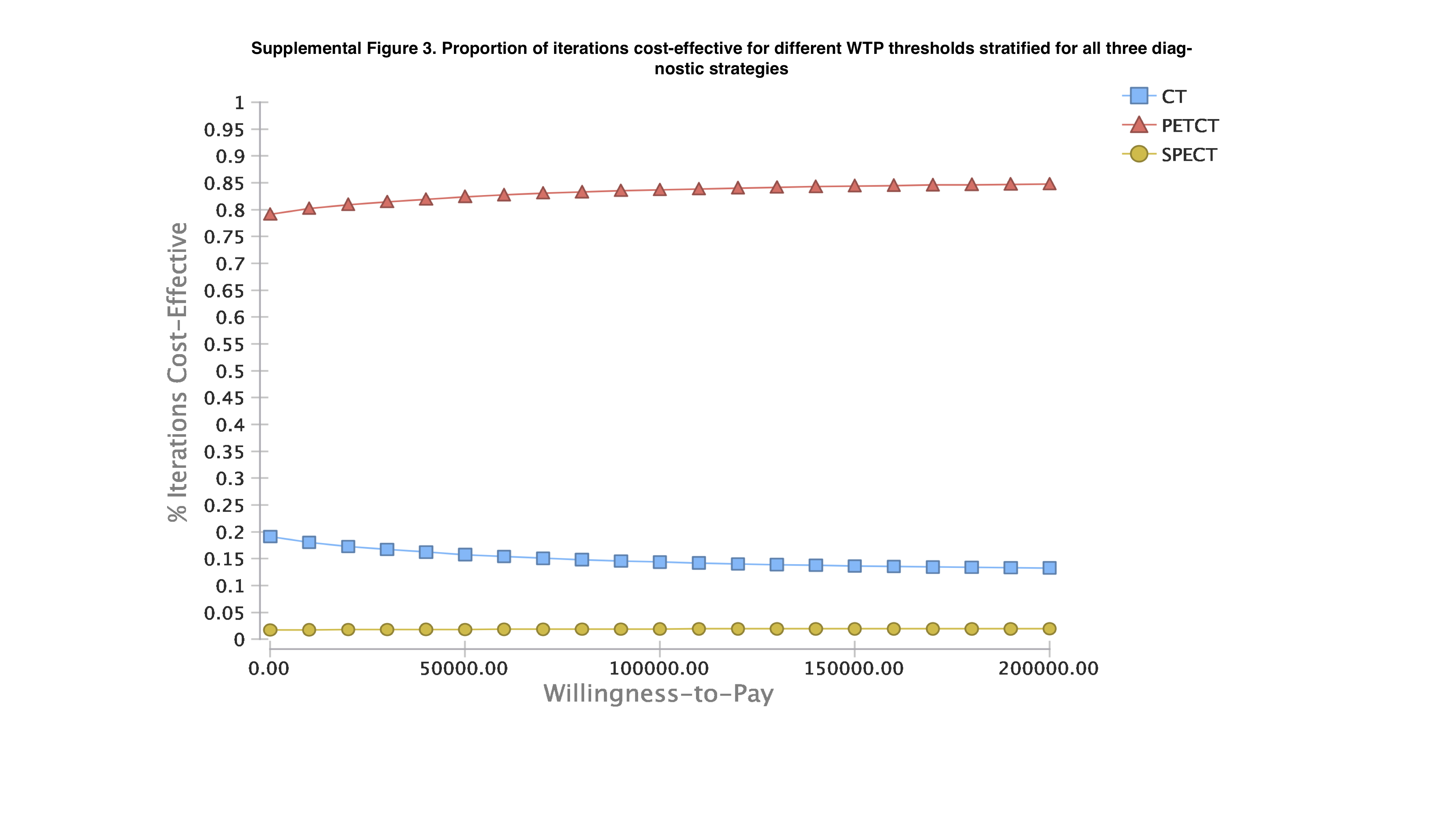

Supplement: Supplementary file 1 [file diagnostics-11-00334-s001.zip › Figure 3 Supplemental NET.tiff]

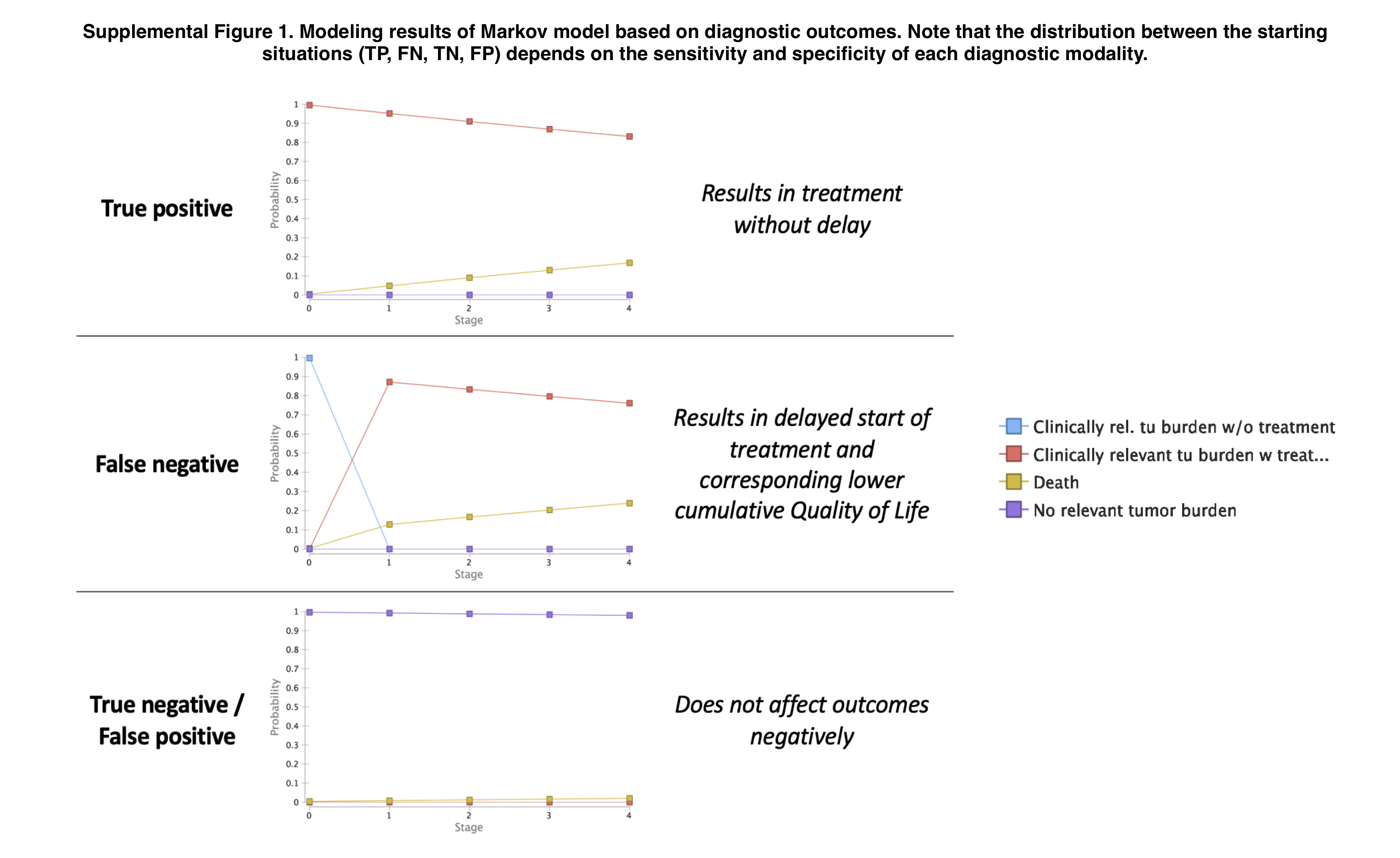

Supplement: Supplementary file 1 [file diagnostics-11-00334-s001.zip › Figure 1 Supplemental NET.tiff]
